# The rate and fate of N_2_ and C fixation by marine diatom-diazotroph symbioses

**DOI:** 10.1038/s41396-021-01086-7

**Published:** 2021-08-24

**Authors:** Rachel A. Foster, Daniela Tienken, Sten Littmann, Martin J. Whitehouse, Marcel M. M. Kuypers, Angelicque E. White

**Affiliations:** 1 Department of Ocean Sciences, University of California, Santa Cruz, CA USA; 2 Department of Biogeochemistry, Max Planck Institute for Marine Microbiology, Bremen, Germany; 3 Department of Ecology, Environment and Plant Sciences, Stockholm University, Stockholm, Sweden; 4 Department of Geosciences, Swedish Museum of Natural History, Stockholm, Sweden; 5 Department of Oceanography, University of Hawai’i at Mānoa, Honolulu, HI USA

**Keywords:** Stable isotope analysis, Microbial ecology, Biooceanography

## Abstract

N_2_ fixation constitutes an important new nitrogen source in the open sea. One group of filamentous N_2_ fixing cyanobacteria (*Richelia intracellularis*, hereafter *Richelia)* form symbiosis with a few genera of diatoms. High rates of N_2_ fixation and carbon (C) fixation have been measured in the presence of diatom-*Richelia* symbioses. However, it is unknown how partners coordinate C fixation and how the symbiont sustains high rates of N_2_ fixation. Here, both the N_2_ and C fixation in wild diatom-*Richelia* populations are reported. Inhibitor experiments designed to inhibit host photosynthesis, resulted in lower estimated growth and depressed C and N_2_ fixation, suggesting that despite the symbionts ability to fix their own C, they must still rely on their respective hosts for C. Single cell analysis indicated that up to 22% of assimilated C in the symbiont is derived from the host, whereas 78–91% of the host N is supplied from their symbionts. A size-dependent relationship is identified where larger cells have higher N_2_ and C fixation, and only N_2_ fixation was light dependent. Using the single cell measures, the N-rich phycosphere surrounding these symbioses was estimated and contributes directly and rapidly to the surface ocean rather than the mesopelagic, even at high estimated sinking velocities (<10 m d^−1^). Several eco-physiological parameters necessary for incorporating symbiotic N_2_ fixing populations into larger basin scale biogeochemical models (i.e., N and C cycles) are provided.

## Introduction

In large expanses of the sunlit open ocean, concentrations of bioavailable nitrogen (N) are low to below analytical detection. In these regions the primary source of new N comes from biological N_2_ fixation (BNF) [1], or the reduction of di-nitrogen (N_2_) to ammonia [2]. Historically, the larger colonial filamentous cyanobacteria, *Trichodesmium* spp., has been considered the dominant N_2_ fixer (diazotroph) [3], whereas recent work has highlighted the equally significant role of smaller unicellular N_2_ fixing cyanobacteria, i.e., *Candidatus* Atelocyanobacterium thalassa (UCYN-A1 and UCYN-A2), which live symbiotically with a eukaryotic alga [4–6]. Seldom is the co-occurring heterocystous symbiotic cyanobacteria *Richelia intracellularis* (hereafter *Richelia*) and *Calothrix rhizosoleniae* (hereafter *Calothrix*) studied or reported in a similar context [7]. Heterocysts are specialized thick-walled cells that are differentiated to isolate the oxygen (O_2_) sensitive nitrogenase enzyme for N_2_ fixation from O_2_ produced in the neighboring photosynthetic vegetative cells [8, 9] (Supplementary Fig. 1). *Richelia* and *Calothrix* are the few heterocystous cyanobacteria commonly found in the oligotrophic oceans, where they form highly specific relationships with eukaryotic diatoms: *Hemiaulus* spp., *Rhizosolenia* spp. and *Chaetoceros compressus*, respectively [10–12].

Cyanobacteria, especially heterocystous types, have a high propensity for symbioses, i.e., the diverse group of land plants (liverworts, hornworts, cycads, ferns, angiosperms, and cyanolichens) that enter partnerships with cyanobacteria [13]. Unlike the marine diatoms, these terrestrial systems are better characterized. A clear distinction in the land plant-cyanobacterial symbioses is the investment by the plant for specialized chambers and cavities to hold their symbionts. In most, the cavities are darkened and therefore the symbiotic cyanobacteria live heterotrophically by reduced C from their respective hosts [14–16]. In many terrestrial examples, the heterocystous cyanobacteria are also intercalary types and differentiate multiple heterocysts along the filament [17]. The heterocysts of *Richelia* and *Calothrix* symbionts are single and terminal, and therefore filament length increases/decreases by changing the number of vegetative cells. After establishment in land plants, symbiotic N_2_ fixation rates increase [18], whereas both C and ammonia assimilation by the symbionts decrease, as does the symbiont growth rate (15–16; 18). Thus, in the terrestrial examples, both partners adapt to the partnership, and in the case of the symbiont, multiple metabolic changes provide direct benefit to their respective hosts.

Draft genomes of the *Richelia* symbiont strains from three diatoms: *H. hauckii* (RintHH01), *H. membranaceus* (RintHM01) and *R. clevei* (RintRC01), are available [19, 20]. The *Calothrix* genome (CalSC01) was also sequenced after its isolation from a chain of *Chaetoceros compressus* diatoms (19; 21). Collectively, the diatom-heterocystous cyanobacterial symbioses are a unique system to study because the symbiont cellular location varies from internal, to “partial”, to fully external (Supplementary Fig. 1). Moreover, the symbiont cellular location is also related to the symbiont genome size, content, and age of the partnerships. For example, the more internal *Richelia* symbionts have smaller draft genomes (RintHM01, 2.21Mbp; RintHH01, 3.24 Mbp) and are more ancient, while external symbionts possess larger genomes (RintRC01, 5.48 Mbp; CalSC01, 5.97 Mbp) and more recently emerged [19–22]. The RintHM01 genome was poorly sequenced and has not been fully analyzed [19], and genomes RintHH01 and RintRC01 are shown to lack several N assimilatory pathways common to cyanobacteria, including N transporters (e.g., ammonium), and N reductases (e.g., nitrate, urease) [19, 20]. Thus, *Richelia*’*s* N assimilation is largely restricted to the energy demanding N_2_ fixation process, whereas the *Calothrix* symbiont draft genome (CalSC01) is more similar in size and content to other free-living heterocystous cyanobacteria [19, 20, 23]. The diversity of the host diatoms is limited to a few phylogenetic markers and two field transcriptomic studies [22, 24, 25].

High rates of N_2_ and C fixation have been measured in bulk water field incubations when *Richelia* symbioses are present [26–29]. Both partners are photosynthetic organisms that possess ribulose 1,5-bisphosphate carboxylase/oxygenase (RubisCO) for carbon (C) fixation [19, 20], yet which partner, if not both, fixes C is unknown. Recently, Nieves-Morión et al. [23] reported the presence and absence of a number of membrane transporters potentially required for metabolic exchanges between the partners. An interesting absence in the RintHH01 and RintRC01 genomes are ABC bicarbonate (HCO_3_^-^; C_i_) transporters common to other cyanobacteria for acquiring inorganic C for CO_2_ fixation [23]. Alternatively, the genomes of the *Richelia* symbionts, encode genes for the NDH-1 type of CO_2_ uptake and genes similar to the SulP-family HCO_3_^-^ transporter BicA [23]. Interestingly, the BicA in RintHH01 is fragmented into four consecutive sequences reflecting incomplete sequencing or gene erosion, whereas in RintRC01 it has remained a full sequence [23]. Hence an open question in the diatom*-Richelia* symbioses is which partner is fixing C and whether the partners compete for CO_2_. In heterocystous cyanobacteria, photosynthesis results in reduced C in the form of sugars which functions as the primary energy source for N_2_ fixation. Recently, a hypothetical flux model based on four metabolic processes (photosynthesis, N_2_ fixation, biosynthesis, respiration) was applied to the *Hemiaulus-Richelia* symbiosis in order to predict how the partners support one another [30]. The model predicted that 25% of the host diatom C fixation was provided to the symbiont to drive N_2_ fixation, and the symbiont supplied a majority (up to 82%) of its fixed N to the host *Hemiaulus* [30]. Hence determining the rate and role of C fixation is important for sustaining the symbioses itself, but also impacts to what extent these symbioses contribute to primary and new production.

Despite their ubiquitous distribution and biogeochemical significance, there are few reported N_2_ and C fixation rate measurements for these symbiotic diatoms [26–29, 31]. In fact, with the exception of a few single cell N_2_ fixation rates reported in Foster et al. [32], all measurements are from bulk and/or cell concentrates (i.e., plankton tows), and therefore include the activities of co-occurring populations or other N_2_-fixers such as *Trichodesmium* spp. and unicellular diazotrophs. In the recent work presented by Pyle et al. [33], rates of N_2_ fixation were estimated on non-axenic enrichment *H. hauckii-Richelia* cultures using indirect acetylene reduction assays. Here, using stable isotope labeling experiments with and without eukaryotic inhibitors and secondary ion mass spectrometry (SIMS) measures, we report a high number of single cell rate measurements of both C and N_2_ fixation for *Hemiaulus*- and *Rhizosolenia-Richelia* symbioses. These represent the largest number of field measurements for these important populations. Because our dataset is from wild populations, we provide several eco-physiological parameters necessary for modeling metabolite exchanges between two partners which have not been well-studied, modeled or understood. These parameters are additionally directly applicable to larger scale models that attempt to predict basin scale N_2_ and C fixation.

## Materials and methods

### Expeditions and sampling

Two expeditions to the western tropical North Atlantic (WTNA) were made between 23 May–21 June 2010 and 9 September–6 October 2011 for sampling and performing the stable isotope incubation experiments described herein (Supplementary Fig. 2; Supplementary Table 1). The stations for incubation experiments were selected based on an initial screening of the surface plankton for the presence of symbiotic diatoms by microscopy (Supplementary Materials). In 2010, stations 2, 4, 5, 9, and 10 had densities of the symbiotic diatoms (>100 cells/filter) deemed suitable for the incubation experiments, while in 2011 cell densities were lower. In 2011, the pre-dawn cast was not always feasible due to time constraints, and only stations 10, 24, and 29 were sampled for incubation experiments for bulk and single cell activity measurements.

### Symbiotic diatom cell counts

Symbiotic cells for microscopy counts were collected directly from the conductivity, temperature, depth (CTD) rosette at the same or similar depths as water collected for the incubation experiments (Supplementary Table 1, Supplementary Fig. 2). Briefly, the entire contents of the Niskin bottles were gravity filtered onto a 47 mm diameter Poretics membrane 10.0 μm pore size filter held within a Swinnex filter holder (Millipore, Billerica, MA USA). Gravity filtration varied from 1 to 2 h (h). If the filter clogged after 2 h, the remaining volume in the Niskin was measured using a graduated cylinder (1 L) and the volume filtered was corrected. The filter was mounted onto an oversized glass slide (75 mm × 50 mm × 1 mm), examined and symbiotic diatoms were identified using blue (450–90 nm) and green (510–60 nm) filter sets on 400 X. The diatoms with *Richelia* were identified as *H. hauckii, H. membranaeus*, and *R. clevei*, based on ultrastructure and size. When symbiotic cells were in high densities (2010 cruise), several smaller grid areas (62.5 µm^2^) of the filter were scanned; otherwise, the whole filter was viewed for counting. Usually, at least 500 symbiotic cells were enumerated, and corrections to the cell abundances were made by the area scanned. The abundances were normalized to the volume filtered and reported as diatom host-*Richelia* L^−1^.

### Incubation experiments

For incubation experiments, whole water was collected from two-six depths at select stations using the CTD rosette (Supplementary Materials; Supplementary Fig. 2; Supplementary Table 1). Whole water was transferred directly into acid-rinsed, transparent, 2.75 L polycarbonate bottles. Bottles were filled without headspace and each bottle amended with 1.5 mL of 0.5 M NaH^13^CO_3_ and 2 mL 99% ^15^N_2_ (Cambridge isotopes, Andover MA USA) through the septa cap using gas tight syringes (Hamilton, Reno, NY USA). The bottles were manually inverted several times to mix the isotopes and placed on their sides in an on-deck incubator with continuously flowing surface seawater. Bottles were incubated during daylight hours (~12 h) and screen shading was used to simulate the 0.1, 1, 25, 50, 75% incident surface irradiance. Water collected from the near surface (0–5 m) were incubated without screening. Surface irradiance was measured as average surface radiation (PAR; µE m^−2^ s^−1^) at the time of sampling using the light sensor mounted on the CTD package. In 2010, due to sampling limitations 1 bottle was collected for whole water bulk analysis, and 1 bottle for SIMS analysis. In 2011, 3 replicate bottles were incubated for whole water bulk analysis and 1 bottle for SIMS analysis. Time points included the time of injection (time 0), and time end (~12 h). At sundown, using a peristaltic pump (Cole-Parmer, Vernon Hills, PA USA), the entire contents of 1 or 3 bottles (2010 or 2011, respectively) were filtered onto a pre-combusted GFF (25 mm diameter) filter held in a Swinnex filter holder for bulk analysis. The filters were placed in sterile Eppendorf tubes (1.5 mL), acid fumed overnight in a desiccator with an open beaker of 37% hydrochloric acid (HCl) to remove inorganic C from the filters, then placed in a 60 °C oven to complete dehydration for 12–24 h. The dried filters were weighed on a microbalance and prepared for combustion analyses in tin capsules.

Measurements of particulate nitrogen (N; µg) and carbon (C; µg) and atom % ^15^N and atom % ^13^C were made with an automated elemental analyzer (Thermo Flash EA, 1112 Series) coupled to a Delta Plus Advantage isotope ratio mass spectrometer (Thermo Finnigan, Dreieich Germany; EA-IRMS). Instrument accuracy and precisions were estimate at 0.3652 ± 0.0002 ^15^N atom % and 1.0660 ± 0.0010 ^13^C atom % based on the mean and standard deviation of caffeine standards measured in conjunction with the samples. Fixation rates were calculated as a function of the change in the tracer concentration of the particulate pool relative to the size of the pool between time 0 and the time end [34].

In 2011, incubations with the eukaryotic inhibitor treatments were set up from populations collected at station 25. Similar procedures as described above were used, but on smaller volume bottles and on concentrated plankton. In these experiments 5 L of surface seawater was collected by bucket and gravity filtered onto a 10 µm (Poretics) pore size 47 mm diameter membrane filter held in a filter tower. The filter was directly placed in a 250 mL acid-washed polycarbonate Nalgene bottle filled with 0.2 µm FSW and capped without air bubbles. When filled without air, these bottles hold 2.75 mL volume. Cycloheximide was amended to a final concentration of 0.05 mg/ml in the treated bottles. No amendments or un-treated bottles were used as the controls; four bottles were set up for controls, and four for cycloheximide. All bottles were amended with 180 μL of 0.5 M NaH^13^CO_3_ and 250 µL of 99% ^15^N_2_ (Cambridge isotopes) in order to have an expected labeling percent of 3% and 10%, respectively. Amendments were made with gas tight syringes as described above and bottles were incubated at 50% incident light. Approximately 10 h after amendments, three whole bottles (2.75 L) were filtered and prepared for the bulk analyses and 1 for the SIMS as described above using a peristaltic pump.

It should be noted that our incubation experiments were performed in 2010 and 2011, which was prior to the extensive evidence demonstrating that N_2_ amendments as a gas result in a slow dissolution and underestimation in N_2_ fixation rates [35–37]. Given the longer incubation times (10–12 h) and the earlier evidence of activity and transfer of fixed N within 30 min to the host diatoms [32] using a similar experimental strategy, we assume that the underestimation in N_2_ fixation was negligible.

### SIMS sampling

At time 0 and time end, 1 bottle (2.75 L or 2.75 mL for inhibitor experiments) was filtered as described above with a peristaltic pump onto a pre-sputtered gold palladium (Au-Pd) 3 µm pore size membrane filter (25 mm diameter; Millipore Type GTTP02500). The cells on the filter were chemically fixed for 1–4 h at room temperature in the dark with 100 µl of 4% paraformaldehyde (PFA; w-v) solution made in 0.2 µm filtered seawater (FSW). Subsequently filters were rinsed three times in 0.1 M phosphate buffered saline (PBS) and stored dry at −20 °C until later analyses.

### Microscopic identification, subsample preparations and markings for SIMS

The Au-Pd filters were initially scanned for the symbiotic diatoms using a Laser Micro Dissection (LMD) microscope 6500 (Leica Wetzlar, Germany) fitted with blue (440-85 nm) and green (517-41 nm) excitation filter sets. The same excitation patterns and morphological features were used as described above for identifying the various symbioses. In 2010 the *H. hauckii-Richelia* were often too dense on the Au-Pd filter, and were re-suspended for dilution. Briefly, the filter was placed in a 2 mL Eppendorf tube with cells facing inwards and amended with 1 mL sterile 0.1 M PBS. Using a 1 mL pipettor, the filter was gently washed multiple times with the PBS. Using small aliquots (100–200 µL), the re-suspended cells were gravity filtered onto a new pre-sputtered Au-Pd (3.0 µm pore size 25 mm diameter) and densities checked. Areas containing the target cells were sub-sectioned into 5 mm diameter spheres, followed by adding laser markings and arrows directly to the filter to easily re-identify areas for analysis during the SIMS. Additional microscopic images were taken for orientation purposes during the SIMS analyses and for post-processing using look@nanoSIMS [38] and WinImage2 (CAMECA) software packages. For example, to identify the numbers and cellular location of symbiont filament in the host diatoms.

In total 45 individual symbiotic cells were analyzed: including 2 *R. clevei-Richelia*, 32 *H. hauckii-Richelia*, 1 *H. membranaceus-Richelia* from 2010 (stations 2, 19, 25) and 10 *H. membranaceus-Richelia* symbioses from 2011 (station 29) (Supplementary Fig. 2). Five of the 10 measurements in 2011 on the *H. membranaceus-Richelia* symbioses were from the eukaryotic inhibitor experiments. These analyses are equivalent to ~32 d (8 h d^−1^) of analyses (includes instrument tuning).

### SIMS analysis

SIMS analyses were performed using a CAMECA NanoSIMS 50 L instrument (Cameca) or a CAMECA ims1280 large-geometry (LG-) SIMS instrument. Briefly, for analyses performed on the NanoSIMS 50 L, each identified cell was pre-sputtered with a special mode developed to burn through the diatom frustules: Cesium (Cs^+^) primary beam between 20–25 nA at D1-0 for 10–15 s. After pre-sputtering, the cells were rastered with a primary ion beam with a current between 1–2 pA and a beam size <100 nm. Mass resolving power (MRP) in all measurements was > 7000. The primary ion beam was used to raster the area of interest at a size between 40 × 40 µm and 50 × 50 µm with an image size of 256 × 256 pixels or 512 × 512 pixels over the chosen raster size with a dwelling time of 1 or 2 ms per pixel. Negative charged secondary ions (^12^C^−^, ^13^C^−^, ^12^C^14^N^−^, ^12^C^15^N^−^, ^31^P^−^, ^32^S^−^) were collected simultaneously in 6 parallel electron multiplier detectors of the multi-collection system of the instrument. All scans (15–200 planes per diatom cell) were corrected for drift of the beam and sample stage and accumulated after acquisition using the look@nanoSIMS software package [38]. The higher number of planes was circumstantial, as occasionally the NanoSIMS 50 L was run overnight and therefore more planes acquired.

Two symbiotic cells (*H. membranceus-Richelia*) from the eukaryotic inhibitor experiments were measured by LG-SIMS. Here the approach was similar, however, an initial 10 × 10 µm larger area than analysis area (50 × 50 µm or 50 × 60 µm) was pre-sputtered for 60 s using ca. 3 nA Cs beam. Subsequently, the analysis region was measured using a ca. 50 pA primary Cs^+^ beam (spot size ~1 µm). The secondary ion mass peaks were measured in peak switching mode in a single ion counting electron multiplier. Count times for the ^12^C^14^N^−^, ^13^C^14^N^−^, ^12^C^15^N ions were 1, 4 and 2 s, respectively. A nominal mass resolution of 7000 (M/ΔM) was used, together with a slight shift of the ^13^C^14^N peak to higher mass (higher effective mass resolution) to avoid a small interference visible on the low mass side of the peak flat (likely ^11^B^16^O).

Isotope ratio images were created as the ratio of the sum of counts for each pixel over all recorded planes of the investigated isotope and the main isotope. Regions of interest (ROIs) were defined using the secondary electron (SE), ^12^C^14^N^−^, and epi-fluorescent images taken prior to the analysis. For each ROI, the isotopic ratio was calculated using WinImage2 (CAMECA) or look@nanoSIMS software packages [38]. The EA-IRMS gives better statistics than the SIMS instruments due to the larger amount of analyzed material and therefore was used to measure the natural abundance of the C and N isotopes in the particulate fraction of the time 0 samples.

The calculations of C and N_2_ fixation rates for the individual symbiotic cells, including all other parameters necessary for the estimations (e.g., biovolume, initial C and N content) and how these values were used to estimate the symbiotic contribution to the bulk activity are described in detail in Foster et al. [32] and again here in Supplementary Materials. The statistical analyses are also summarized in Supplementary Materials.

## Results and discussion

### Abundances of N_2_ fixing symbioses in the WTNA

To date, the various marine symbiotic diatoms are notoriously understudied, and hence our understanding of their abundances and distribution patterns is limited [7]. In general, these symbiotic populations are capable of forming expansive blooms, but largely co-occur at low densities in tropical and subtropical waters with a few rare reports in temperate waters [26–29, 39–42]. The *Rhizosolenia-Richelia* symbioses have been more commonly reported in the North Pacific gyre [26, 27, 31], and the western tropical North Atlantic (WTNA) near the Amazon and Orinoco River plumes is an area where widespread blooms of the *H. hauckii-Richelia* symbioses are consistently recorded [28, 29, 42–47].

In the summer of 2010, bloom densities (10^5^−10^6^ cells L^−1^) of the *H. hauckii-Richelia* symbioses were encountered at multiple stations with mesohaline (30–35 PSU) surface salinities (Supplementary Table 1). The *R. clevei-Richelia* symbioses were less abundant (2–30 cells L^−1^). Similar densities of *H. hauckii-Richelia* have been reported in the WTNA during spring (April–May) and summer seasons (June–July) (28–29; 46). In fall 2011, less dense symbiotic populations (0–50 cells L^−1^) were observed, and the dominant symbioses was the larger cell diameter (30–50 µm) *H. membranaceus* associated with *Richelia*. Previous observations of *H. membranaeus-Richelia* in this region are limited and reported as total cells (i.e., 12-218 cells) and highest numbers recorded in Aug–Sept in waters near the Bahama Islands [43]. On the other hand, *Rhizosolenia-Richelia* are even less reported in the WTNA, and most studies by quantitative PCR assays based on the *nifH* gene (for nitrogenase enzyme for N_2_ fixation) of the symbiont (44; 46–7). Unlike qPCR which cannot resolve if the populations are symbiotic or active for N_2_ fixation, the densities and activity reported here represent quantitative counts and measures of activity for symbiotic *Rhizosolenia*.

The WTNA is largely influenced by both riverine and atmospheric dust deposition (e.g., Saharan dust) [48], including the silica necessary for the host diatom frustules, and trace metals (e.g., iron) necessary for photosynthesis by both partners and the nitrogenase enzyme (for N_2_ fixation) of the symbiont. We observed similar hydrographic conditions (i.e., low to immeasurable concentrations of dissolved N, sufficient concentrations of dissolved inorganic P and silicates, and variable surface salinities; 22; 28–29; 40–47) as reported earlier that favor high densities of *H. hauckii-Richelia* blooms. Unfortunately our data is too sparse to determine if these conditions are in fact priming and favoring the observed blooms of the *H.hauckii-Richelia* symbioses in summer 2010, and to a lesser extent in the Fall 2011.

### A biometric relationship between C and N activity and host biovolume

The diatom-*Richelia* symbioses are considered highly host specific [10, 11], however, the driver of the specificity between partners remains unknown. We initially hypothesized that host selectivity could be related to the N_2_ fixation capacity of the symbiont. Moreover, it would be expected that the larger *H. membranaceus* and *R. clevei* hosts which are ~2–2.5 and 3.5–5 times, respectively, larger in cell dimensions than the *H. hauckii* cells would have higher N requirements (Supplementary Table 2). In fact, recently it was reported that the filament length of *Richelia* is positively correlated with the diameter of their respective hosts [22]. Thus, to determine if there is also a size dependent relationship between activity and cell biovolume, the enrichment of both ^15^N and ^13^C measured by SIMS was plotted as a function of symbiotic cell biovolume.

**Fig. 1:**
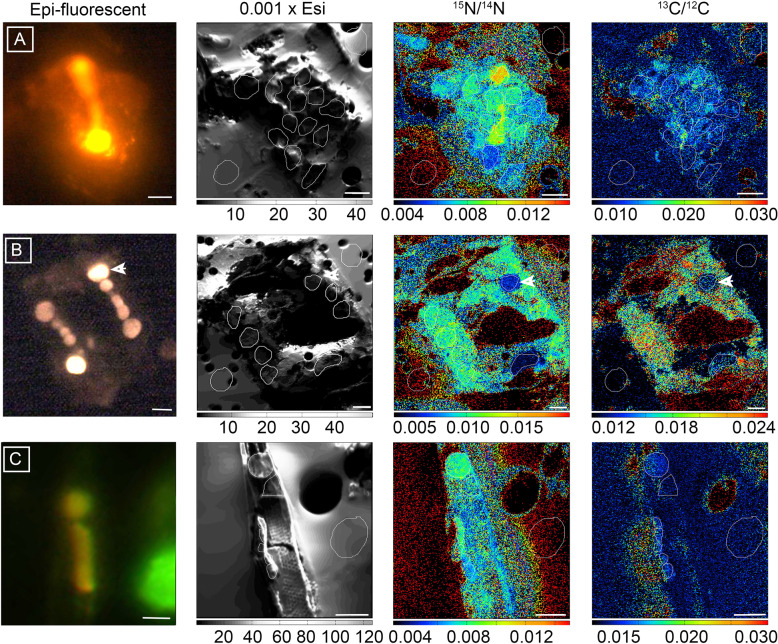
Representative epi-fluorescent micrographs and NanoSIMS imaging of wild collected symbiotic diatom-*Richelia* symbioses incubated with ^15^N_2_ and ^13^C-bicarbonate for 12 h under in situ conditions. Images from left to right include: epi-fluorescent image taken prior to NanoSIMS analyses and correspond to the parallel NanoSIMS imaging of total secondary ion count (0.001 x Esi), enrichment of ^15^N (^15^N/^14^N), and enrichment of ^13^C (^13^C/^12^C). The epi-fluorescent images show excitation patterns expected for the symbiont and host chloroplast. The secondary ion content images show the host cellular boundaries and destructive nature of the NanoSIMS analyses. (**A**) Symbiotic *H. hauckii-Richelia* cell collected from 2 m with 2 *Richelia* filaments emitting a bright orange-red fluorescence under green excitation (510–60 nm). The strong fluorescence associated with the *Richelia* filaments corresponds to a high ^15^N enrichment (^15^N/^14^N ratio image), whereas cellular ^13^C enrichment (^13^C/^12^C) is above background but uniformly low. (**B**) A larger *H. membranaceus-Richelia* cell collected from 25 m with two clearly fluorescent filaments of *Richelia*. Note the terminal heterocysts on either end of the two filaments, indicating recent or in situ cellular division of the *Richelia*. NanoSIMS images show uniform high enrichment of both ^15^N and ^13^C (^15^N/^14^N and ^13^C/^12^C ratio images respectively) in areas of both symbiont and host chloroplast with the exception of one heterocyst designated with an arrow. (**C)** The blue excitation (459–90 nm) micrograph of the apical end of a symbiotic *R. clevei-Richelia* cell shows a fluorescent yellow *Richelia* and corresponds to high ^15^N enrichment (^15^N/^14^N ratio image) in the heterocyst whereas the corresponding ^13^C enrichment (^13^C/^12^C ratio image) is low. Here, the secondary ion content image distinguishes the remnants of the diatom frustule. The enclosed markings in the NanoSIMS images define the regions of interest (ROIs), which were used to determine the ^13^C/^12^C and ^15^N/^14^N ratios. Scale bars are 5 μm.

**Fig. 2:**
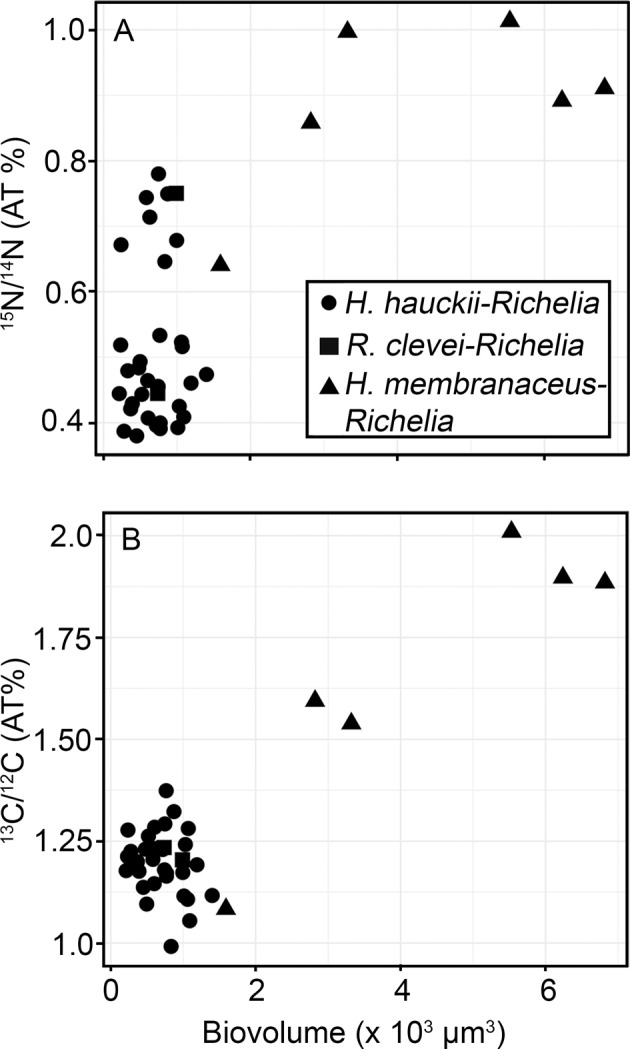
Biometric relationship between isotopic enrichment and biovolume (µm^3^). (**A**) ^15^N and (**B**) ^13^C; enrichment reported as atom percent (AT%).

Given the long incubation times (12 h) and previous work [32] that show fixation and transfer of reduced N to the host is rapid (i.e., within 30 min), we expected most if not all of the reduced N, or enrichment of ^15^N, to be transferred to the host diatom during the experiment (Fig. 1). Therefore, we measured and report the enrichment for the whole symbiotic cell, rather than the enrichment in the individual partners (Supplementary Table 2; Fig. 2). The enrichment of both ^13^C/^12^C and ^15^N/^14^N was significantly higher in the larger *H. membranaceus-Richelia* cells (atom % ^13^C: 1.5628–2.0500; atom % ^15^N: 0.8645–1.0200) than the enrichment measured in the smaller *H. hauckii-Richelia* cells (atom % ^13^C: 1.0700–1.3078; atom % ^15^N: 0.3642–0.7925) (Fig. 2) (^13^C, Mann–Whitney *p* = 0.009; ^15^N, Mann–Whitney *p* < 0.001). In addition, the higher enrichment also corresponded to higher rates of N_2_ fixation (e.g., 12.6–28.4 fmol N cell^−1^ h^−1^ for *H. membranaceus-Richelia* compared to 0.1-4.29 fmol N cell^−1^ h^−1^ for *H. hauckii-Richelia;* Supplementary Table 2) and C fixation (219–685 fmol C cell^−1^ h^−1^ compared to 2.74–59.8 fmol C cell^−1^ h^−1^, respectively; Supplementary Table 2).

Similar findings of higher ^15^N enrichment and N_2_ fixation rates were also reported by Martínez-Pérez et al. [5], when comparing the larger N_2_ fixing UCYN-A2-haptophyte symbioses to the smaller UCYN-A1-haptophyte symbiotic cells. Here, however, pooling all the SIMS measurements and biovolume estimates for the three different symbioses, we present a robust relationship between enrichment (atom % ^13^C or atom % ^15^N) and biovolume for both C and N (Fig. 2). It suggests that the larger size demands a higher activity for both N and C, which would be expected from allometric theory. Here, allometry refers to the relationship that biological processes scale by body-size [49].

### Light dependence of N_2_ fixation by diatom-*Richelia* symbioses

In order to determine if metabolic activity for the various symbioses is light dependent, the stable isotope incubation experiments were set up under simulated light conditions of the water column (i.e., 0.1−100% incidence surface light). Activity rates are reported for both bulk (see below) and single cells.

Although the data is more limited for the two larger symbiotic diatoms: *H. membranaceus* and *R.clevei-Richelia* symbioses, both follow the expected trend for phototrophic organisms to have higher C fixation in cells collected nearer to the surface and incubated under higher light intensities [50]. For example, the *H. membranaceus-Richelia* symbioses collected nearer to the surface had higher ^13^C enrichment and rates of C fixation than those collected from a deeper depth (25 m). However, ^15^N enrichment and corresponding N_2_ fixation rates for the *H. membranaceus-Richelia* symbioses were similar regardless of their depth of collection. The two *R. clevei*-*Richelia* symbioses measured high and comparable enrichment of ^13^C and to a lesser extent ^15^N (atom % ^13^C = 1.2053 and 1.2346; atom % ^15^N = 0.7543 and 0.4480) to that measured in the *H. membranaceus-Richelia* symbioses (Supplementary Table 2).

**Fig. 3:**
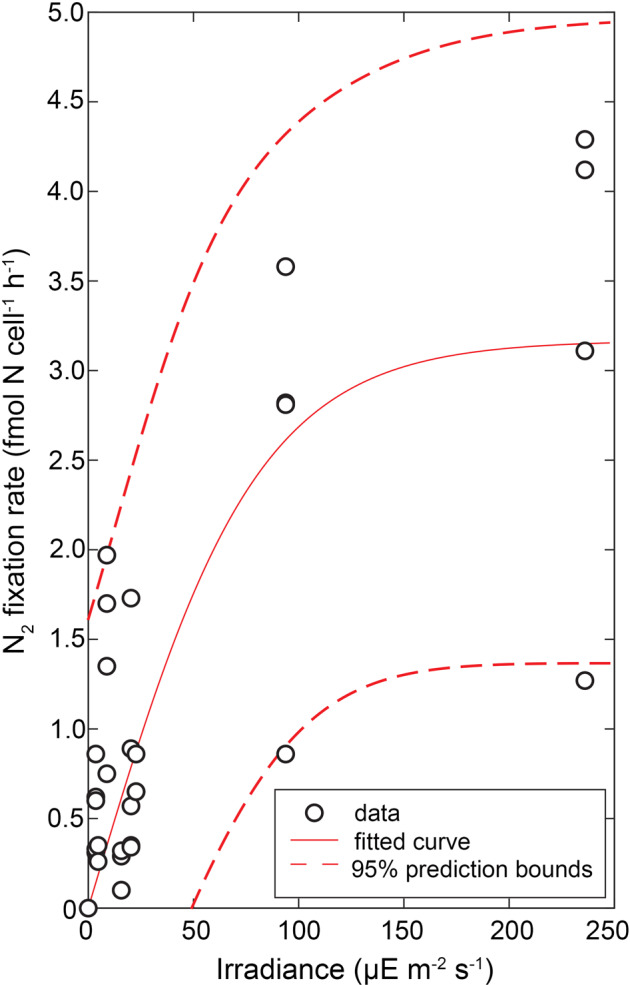
The sensitivity of ^15^N_2_ fixation activity to light for the *H*. *hauckii-Richelia* symbioses. Irradiance values reflect that measured at the time of collection and simulated during incubation. A hyperbolic tangent model [33] was used to fit the data and estimate (solid line) the maximum rate of N_2_ fixation (NFR_max_, 3.1 fmol N cell^−1^ h^−1^) and the initial slope of the rate-response curve (α_NFR_, 0.044 fmol N cell^−1^ h^−1^). The 95 % confidence intervals for the model fit is shown in dashed red lines and correspond to lower and upper bounds of NFR_max_ of 2.3-3.9 fmol N cell^−1^ h^−1^ and α_NFR_ of 0.018–0.07 fmol N cell^−1^ h^−1^. The adjusted *R*^2^ = 0.56.

Given the higher number of measurements for the *H. hauckii-Richelia* symbioses at multiple light levels, we plotted the individual rates of N_2_ and C fixation as a function of photosynthetically active radiation (PAR) measured at the time of collection (Fig. 3). A hyperbolic tangent model [33] fit well for the N_2_ fixation rate (adjusted *R*^2^ = 0.56). This indicates that N_2_ fixation followed a light dependence and saturation kinetics of maximum N_2_ fixation (N_2_fix_max_) and enrichment (^15^N_max_) at the highest irradiances. Unexpectedly, the light response curve for C fixation could not be fit with the same or any saturation model. In fact, the ^13^C enrichment and corresponding estimated C fixation rates were highly variable (Supplementary Table 2). N_2_ fixation in heterocystous cyanobacteria is fueled by C fixation and thus one expects parallel trends in activity for C and N_2_ fixation [51]. We hypothesize that the fixed C of both partners is partitioned into growth and respiration to fuel other metabolic activities (i.e., N_2_ fixation), and resulted in highly variable enrichment patterns. The estimated N- and C- based growth rates (see below) indicated that the cells were also growing at different rates and additionally could influence the observed variation. Further experimentation would be required to test these hypotheses and both require shorter incubations and multiple time points than presented here.

Similar lab experiments for determining the light dependency on N_2_ fixation have been reported in the facultative *Calothrix* SC01 strain that forms symbioses with *Chaetoceros* diatoms, in a few older lab studies of *R. clevei-Richelia* and a recent report on *H. hauckii-Richelia* enrichment cultures [21, 33, 52, 53]. In these earlier works, the same light dependent N_2_ fixation is shown. The strong light dependent activity shown here, however, was somewhat surprising given that measures were derived from field populations, unlike these other experiments which were done in controlled laboratory settings and additionally *Calothrix* SC01 was growing asymbiotic (21; 52–53). Furthermore, our measurements are pooled from three different stations with experiments that span approximately four weeks and two stations ~700 km apart (Supplementary Fig. 2). The wild populations studied here were likely different subpopulations and/or at different stages of their growth cycle, and still the response in their N_2_ activity fit well to the curve. In fact, N and C-based growth rates (Supplementary Table 2) identified significant differences in estimated growth rates between the cells collected at the three stations (C-based growth rate: Kruskal–Wallis, *p* = 0.09; N-based growth rate: Kruskal–Wallis, *p* = 0.004). These growth rates were calculated from the SIMS derived cellular ^13^C/^15^N enrichment and initial N/C content based on biovolume (see Supplementary Materials). Moreover, both N-based and C-based growth was depth dependent, where cells nearer to the surface (stations 2 and 19) had higher estimated growth rates (Supplementary Table 2; Kruskal–Wallis, *p* = 0.002 and *p* = 0.008, respectively). Hence, genetically distinct symbiotic strains have a similarly robust N_2_ fixing activity response to light despite varying growth states.

Increased N_2_ fixation by symbiotic cyanobacteria is largely under the host control in many terrestrial symbioses (17; 54). A similar scenario was suggested in the early work of applying NanoSIMS to field collected symbiotic diatoms because higher rates of N_2_ fixation were reported in the symbiotic populations compared to asymbiotic ones [32]. However, in terrestrial symbiotic examples, the number of symbionts per host is dramatically different. Typically, one to two *Richelia* filaments associate with a *Hemiaulus* spp. host, whereas in plants, a symbiotic chamber houses 100’s to 1000’s of symbionts [17, 54]. Moreover, in most terrestrial examples, the number of heterocysts per symbiont filament increases after establishment [17, 18], however in *Richelia* (and *Calothrix*), the heterocysts are single and terminal. Thus, *Richelia* sustains a high N_2_ fixing capacity by maintaining short filaments and a high heterocyst to vegetative cell ratio [23]. It is plausible and hypothesized that *Richelia* acquires energy from their hosts, e.g., in the form of C substrates. This would be a particularly attractive strategy for the internal *Richelia* symbionts of *Hemiaulus* spp. because they reside in close proximity to the host photosynthetic machinery [22].

### Reduced C and N_2_ fixation when host is inhibited

To better understand the potential control and role of C fixation mediated by the host diatoms, ^15^N_2_ and ^13^C-bicarbonate incubation experiments were treated with cycloheximide, a eukaryotic cytosolic protein translation inhibitor. Here, the enrichment of ^15^N and ^13^C were visualized and measured by SIMS in both the symbiont and the host separately. DCMU (3-(3,4-dichlorophenyl)−1, 1-dimethylurea) and chloramphenicol are other common inhibitors, however, both have been shown to inhibit several cyanobacteria strains, including heterocystous types [55]. Cycloheximide is not inhibitory to cyanobacteria and often applied as a cultivation strategy to avoid enrichment of eukaryotes [56]. Earlier lab studies on diatoms (and other eukaryotic algae, i.e., dinoflagellates) have shown that at similar concentrations (0.1–10 μg/ml) of cycloheximide, as used here, can reduce key metabolic processes, including photosynthesis and energy generation [57–59]. Hence, cycloheximide seemed to be an attractive and appropriate inhibitor for shutting down the diatom host photosynthesis, because the cyanobacterial symbiont would not be influenced.

**Fig. 4:**
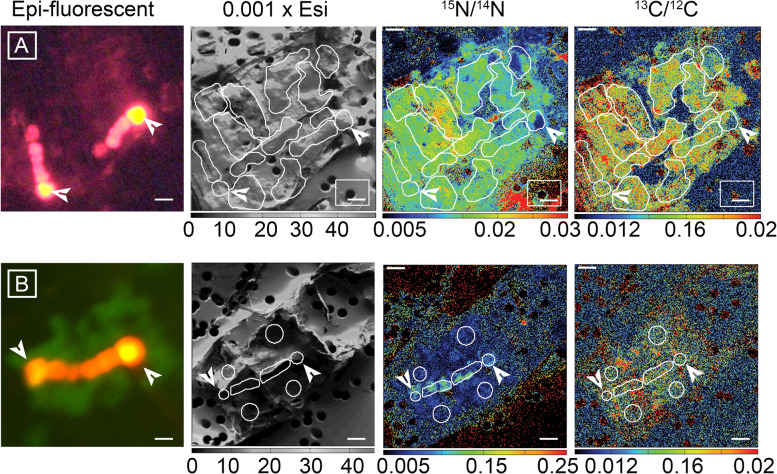
Paired micrographs and NanoSIMS images of ^13^C and ^15^N distribution in *Hemiaulus-Richelia* symbioses, which were incubated with ^13^C-bicarbonate and ^15^N_2_ and untreated (**A**) or treated with a eukaryotic inhibitor (**B**). Images from left to right include: Epi-fluorescent image taken prior to NanoSIMS analyses and correspond to the parallel NanoSIMS imaging of total secondary ion count (0.001 x Esi), enrichment of ^15^N (^15^N/^14^N), and enrichment of ^13^C (^13^C/^12^C). Epi-fluorescent images (**A** panel, green excitation: 510–60 nm; **B** panel, blue excitation: 459–90 nm) taken prior to analyses are used to approximate the cellular location of the symbiont and host chloroplast. The emission visible in the epi-fluorescent micrographs correspond to the filaments of *Richelia* and chloroplast of the host diatoms. Each host cell has two symbiotic filaments, and the white arrows designate the terminal heterocysts of the *Richelia* filaments, which emit red and yellow-orange under green (**A**) and blue excitation (**B**), respectively. Note that in the control cells (untreated, **A** or top images), enrichment of ^15^N and ^13^C is uniformly high in both host and symbiont, whereas the cell treated with eukaryotic inhibitor has localized ^15^N enrichment to the two symbiont filaments and generally low enrichment of ^13^C in whole cell (**B**, or bottom images). The enclosed markings in the NanoSIMS images define the regions of interest (ROIs), which were used to determine the ^13^C/^12^C and ^15^N/^14^N ratios. Scale bars are 5 μm.

The enrichment of both ^15^N/^14^N and ^13^C/^12^C was significantly reduced in the inhibited cells (Fig. 4; Supplementary Table 3; *T* test; *p* < 0.03). The decreased enrichment was measured in the host cells and only the heterocysts of *Richelia* filaments compared to the respective control cells. In fact, in the inhibited cells, the enrichment for ^15^N/^14^N was clearly localized to the symbiont filament, whereas ^13^C/^12^C was reduced overall without an apparent localization (Fig. 4B). The average decrease in ^13^C/^12^C and ^15^N/^14^N enrichment was one to two times, or 16–17% and 36–46% reduced in the hosts and heterocysts of inhibited cells, respectively. The reduction in enrichment resulted in a 66–78% and 76% reduction in estimated rates of C and N_2_ fixation, respectively, when the cells were inhibited. These results are congruent with studies on terrestrial-based symbioses, where the CO_2_ (and NH_4 _^+ ^) assimilation rates are measurably depressed in symbiotic cyanobacteria (17–18; 54). Increased N_2_ fixation is expected when the host is present, as it is the basis of the partnership and has been previously reported [32]. Moreover, it could be that the increased C fixation by the host leads to C:N ratio favoring increased N_2_ fixation. The latter hypothesis remains to be tested.

The reduction in both C and N_2_ fixation in inhibited cells indicates that the host is required, and likely controls the symbiont’s metabolism. In heterocystous cyanobacteria, there is a coordinated effort between vegetative cells and heterocysts to exchange energy, organic C and fixed N. Reduced C is rapidly transported to the heterocysts from vegetative cells, and N_2_ fixed in heterocysts is exported to vegetative cells [60–62]. The ^13^C/^12^C of vegetative cells was similar between inhibited and control cells (*t* test, *p* = 0.08); hence C fixation by *Richelia* persisted in the absence of the host.

The ^13^C/^12^C in terminal heterocysts of untreated cells was, however, significantly higher than the inhibited cells indicating that the host must also contribute to the increased ^13^C/^12^C enrichment measured in the heterocysts. One plausible scenario could be that reduced C substrates derived from the host photosynthesis are transferred to the *Richelia* vegetative cells and subsequently transported to the heterocysts. Encoded in the *Richelia* RintHH01 genome is an invertase (InvB), an enzyme used to irreversibly split sugar [23]. In *Anabaena* sp. 7120, a related heterocystous cyanobacteria, InvB is heterocyst specific and sucrose functions as a reduced C substrate for heterocysts that is transported from vegetative cells [63, 64]. Unfortunately, our incubation period was too long to clearly resolve the transfer of C from the host to symbiont. Shorter time incubations akin to a pulse-chase experiments would be desirable for estimating the gradual transfer, or perhaps a dual label incubation experiment of ^14^C- and ^13^C-labels combined with NanoSIMS to trace the fate of labeled carbon [65].

Using the quantitative measures by SIMS in the control cells, we estimate that 8–22% of assimilated ^13^C in the symbiont is derived from the host, and 78–91% of the host ^15^N is derived from the symbiont. Our estimates are consistent with a recent cellular model of *Hemiaulus-Richelia* that reported 25% of fixed C in the symbiont is derived from the host, and 82% of fixed N in the host is from the symbiont [30]. In the cellular model, however, input values were largely derived from literature values of biovolume, biovolume to C relationships, and assumed stoichiometry [30]. The variation we measured here in the host derived C (8–22%) could be attributed to our long incubation times and thus “bottle effects” [66]. It is also likely that some fixed C was respired in order to fuel N_2_ fixation. Moreover, we have little knowledge of the life history of the cells prior to collection, hence we cannot discount that the population of cells were in varying growth states and contributed to the observed variation. In fact, the estimated growth (both N- and C- based) was higher in the control cells compared to the inhibited cells (*p* = 0.009). The latter is expected because activity was largely inhibited in the treated cells. However, in both the control and inhibited cells, the symbiont (vegetative cells) had higher estimated growth than the respective host cell (Supplementary Table 3; *p* = 0.02). In plant-cyanobacteria symbioses, slower growth is reported in the symbionts [18]; hence our results are unexpected and suggest uncoordinated growth cycles between the partners.

### Impact of symbiotic diatoms on bulk N_2_ and C fixation

Studies on rate and fate of N_2_ and C fixation by the symbiotic diatoms are fewer compared to investigations on other N_2_ fixers i.e., *Trichodesmium*, unicellular diazotrophs. In fact, searching the open world data archive PANGEA for datasets reported for the latter groups, and *Richelia* finds 1564, 126, and 35 datasets, respectively. Thus, the data presented here and motivation for the work was to contribute new rate information for these biogeochemical relevant yet under-reported populations.

To date, there are a few estimates of N_2_ fixation by the *Hemiaulus-Richelia* populations from the same WTNA region. These earlier studies attribute a large source of new N to the photic zone derived from these symbiotic diatom populations [28, 29, 67, 68]. Moreover, this new N contributes significantly to C export in the region [29, 67, 68] and likewise in other regions where diatom-*Richelia* symbioses persist (i.e., N. Pacific: 26–27; 31). However, the previous measurements of N_2_ fixation in the WTNA were indirect because acetylene reduction assays were used and applied to cell concentrates (i.e., plankton “slurries”). Thus, the individual cell activity cannot be distinguished. Combining the single cell measurements by SIMS with cell abundances by microscopy (Supplementary Table 1, 2) we could more accurately estimate the fraction of N_2_-fixation attributable to the symbiotic populations.

It is important to note that although our measures are on individual cells, the populations were incubated in whole water bottle experiments. Hence, we cannot discount that some enrichment in the individual cells is derived from the transfer of reduced substrates from co-occurring populations of other N_2_ fixers. In 2010, however, incubations were performed at stations almost entirely dominated by *H.hauckii-Richelia* cells (>10^4^ cells L^−1^), whereas in 2011, biomass in general was low, and observations of other N_2_ fixers i.e., *Trichodesmium* spp.*, Crocosphaera watsonii*, were rare in general and in particular at the stations assayed for N_2_ and C fixation measurements.

In 2010, we estimate that between 12–53% of the bulk N_2_ fixation was accounted for by the *H. hauckii-Richelia* populations. Although the symbiotic cell densities at depth (11–21 m) of station 2 were slightly higher than the populations in the surface (2–4 m), the surface *H. hauckii-Richelia* cells had higher individual N_2_ fixation rates (0.86–3.58 fmol N cell^−1^ h^−1^) and therefore made a larger contribution (53%) to the total fixed N_2_. Meanwhile, cell densities at station 25 (10^5^ cells L^−1^) were similar to station 2, however rates of N_2_ fixation (0.10–0.35 fmol N cell^−1^ h^−1^) were reduced, and therefore the estimated contribution (12–17%) to bulk N_2_ fixation was lower at station 25. The latter findings are directly relevant to models that use abundance estimates (including mRNA abundances of *nifH* genes for nitrogenase), rather than in situ activity to estimate new N contributing to N budgets [e.g., 69, 70].

The contribution to total C fixation by the symbioses was estimated to be far less than their N contribution. For example, *H. hauckii-Richelia* accounts for only 2–5% of bulk C fixation at stations 2 and 25, and is comparatively less than the contribution of 12–53% to bulk N_2_ fixation. Observations of the *H. hauckii-Richelia* populations during the bloom reported that one or both partners possessed variable cell integrity [46]. For example, long diatom chains (8–12 symbiotic cells per chain and >50 symbiotic cells in a chain) were reported at station 2 with fully intact symbiotic *Richelia* filaments (2–3 vegetative cells and terminal heterocyst), and at station 25 chains were short (1–2 symbiotic cells) and associated with short *Richelia* filaments (only terminal heterocyst). Moreover, the symbiotic *H. hauckii* hosts possessed poor chloroplast auto-fluorescence at station 25 [46]. Given that the cells selected for NanoSIMS were largely single cells, rather than chains, we suspect that these cells were in a less than optimal cell state, which was also reflected in the low ^13^C/^12^C enrichment ratios and low estimated C-based growth rates (0.30–57 div d^−1^). These are particularly reduced compared to the growth rates recently reported for enrichment cultures of *H. hauckii-Richelia* (0.74–93 div d^−1§^) (Supplementary Table 2) [33].

In 2011, higher cellular N_2_ fixation rates (15.4–27.2 fmols N cell^−1^ h^−1^) were measured for the large cell diameter *H. membranaceus-Richelia*, symbioses. Despite high rates of fixation, cell abundances were low (4–19 cells L^−1^), and resulted in a low overall contribution of the symbiotic diatoms to the whole water N_2_ (>1%) and C-fixation (>0.01%). The estimated C-based growth rates for *H. membranaceus* were high (1.9–3.5 div d^−1^), whereas estimated N-based growth rates (0.3–4 div d^−1^) were lower than previously published (33; 52–53). Hence the populations in 2011 were likely in a pre-bloom condition given the low cell densities.

### Estimating symbiotically derived reduced N to surface ocean

To date, determining the fate of the newly fixed N from these highly active but fragile symbiotic populations has been difficult. Thus, we attempted to estimate the excess N fixed and potentially available for release to the surround by using the numerous single cell-specific rates of N_2_ fixation determined by SIMS on the *Hemiaulus* spp.*-Richelia* symbioses (Supplementary Materials). Because the populations form chains during blooms and additionally sink, we calculated the size-dependent sinking rates for both single cells and chains (>50 cells). Initially we hypothesized that sinking rates of the symbiotic associations would be more rapid than the N excretion rates, such that most newly fixed N would contribute less to the upper water column (sunlit).

**Fig. 5:**
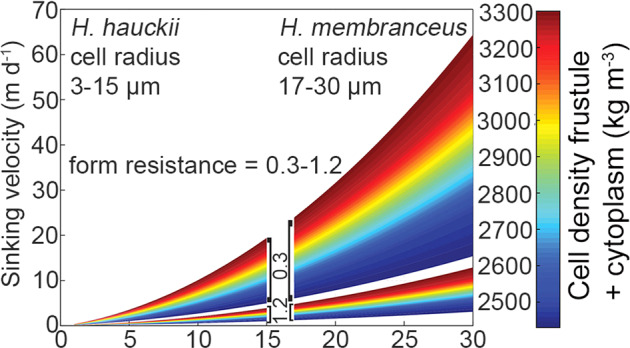
The influence of cell characteristics on estimated sinking velocity for symbiotic *Hemiaulus* spp. The range of diatom sinking speed predicted using the modified Stokes approximation for diatoms [74] and accounting for the symbioses (cylinders) having varying cell size characteristics (form resistance by altering chain length, density; Supplementary Table 4). Note that form resistance increases with chain length and that the longest chains would have sinking speeds less than 10 m d^−1^.

The sinking velocities were plotted (Fig. 5) as a function of cell radius at a range (min, max) of densities and included two different form resistances (∅ = 0.3 and 1.5). As expected, the combination of form resistance and density has a large impact on the sinking velocity. For example, a *H. hauckii* cell of similar radius (10 μm) and density (3300 kg m^−3^) but higher form resistance (0.3 vs. 1.5) sinks twice as fast at the lower form resistance (Fig. 5). This points to chain formation (e.g., increased form resistance) as a potential ecological adaptation to reduce sinking rates. Recently, colony formation was identified as an important phenotypic trait that could be traced back ancestrally amongst both free-living and symbiotic diatoms that presumably functions for maintaining buoyancy and enhancing light capture [22].

**Fig. 6:**
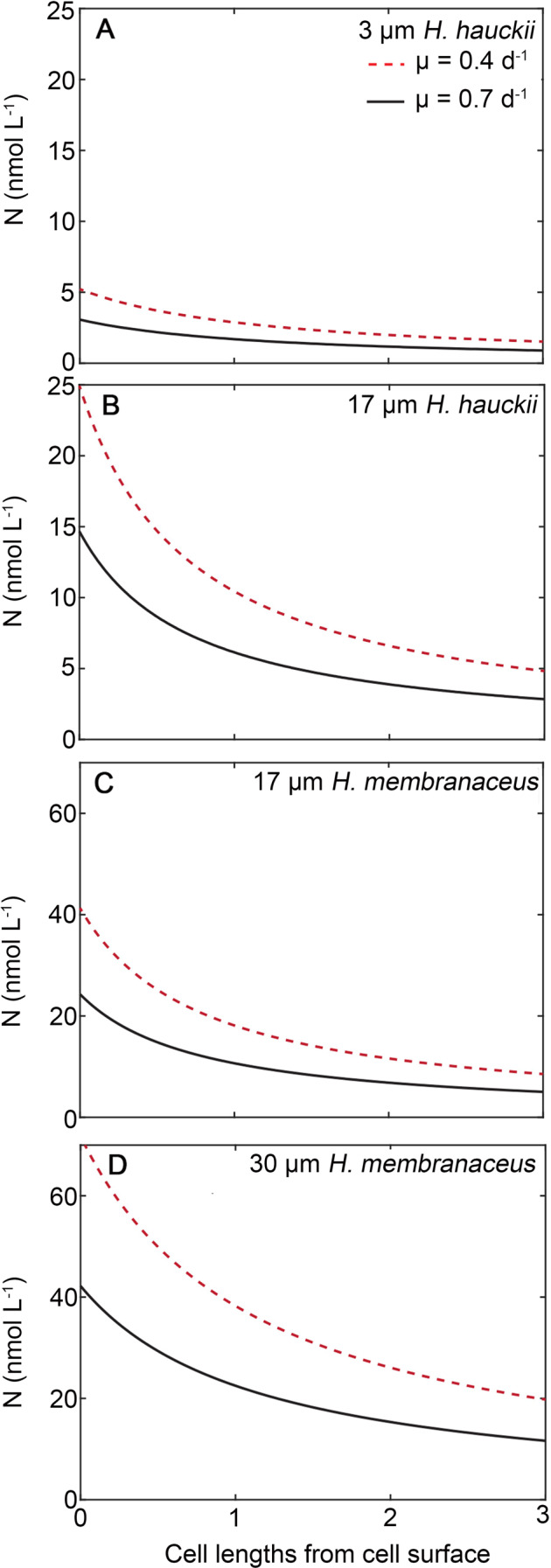
The simplified case of diffusive nitrogen (N) exudate plumes for non-motile symbioses. The concentration of dissolved N (nmol L^−1^) is presented at of varying cell sizes (3 µm and 30 µm) for *H. hauckii-Richelia* (**A** and **B**, respectively) and *H. membranaceus-Richelia* (**C** and **D**, respectively) growing at specific growth rates of 0.4 d^−1^ (dashed red lines) or 0.68 d^−1^ (solid black lines). Exudation follows the same principle as diffusive uptake as per Kiorboe [72] in the absence of turbulence.

The concentration of fixed N surrounding a *H. hauckii* and *H. membranaceus* cell were modeled (Supplementary Materials; Supplementary Table 4; Fig. 6). First, the cellular N requirement (Q_N_, mol N cell^−1^) for a cell of known volume, V, as per the allometric formulation of Menden-Deuer and Lessard [71] is calculated by the following.

1 $${{{{{{{\mathrm{Q}}}}}}}}_{{{{{{{\mathrm{N}}}}}}}} = (10^{ - 12}/12) \times 0.76 \;\times\, {{{{{{{\mathrm{V}}}}}}}}^{^{0.189}}$$

Volume calculations assume a cylindrical shape; whereas exudation assumes an equivalent spherical volume. Then, using published growth rates of 0.4 d^−1^ and 0.68 d^−1^ for the symbioses [52, 53], N uptake rate (V_N_) necessary to sustain the Q_N_ was determined. N loss was assumed to be a constant fraction (f) of the V_N_; this fraction was assumed to be 7.5% and 11% for *H. hauckii* and *H. membranaceus*, respectively, or the estimated excess N which was fixed given the assumed growth rate [31]. The excretion rate (E_N_) of the individual cells was then calculated as

2 $${{{{{{{\mathrm{E}}}}}}}}_{{{{{{{\mathrm{N}}}}}}}} = {{{{{{{\mathrm{fQ}}}}}}}}_{{{{{{{\mathrm{N}}}}}}}}$$

The concentration of fixed N surrounding the cell (C_r_) was iteratively calculated by the following:

3 $${{{{{{{\mathrm{C}}}}}}}}_{{{{{{{\mathrm{r}}}}}}}} = {{{{{{{\mathrm{E}}}}}}}}_{{{{{{{\mathrm{N}}}}}}}}/(4\pi * {{{{{\mathrm{D}}}}}}* {{{{{\mathrm{r}}}}}}_{{{{{\mathrm{{x}}}}}}}) + {{{{{{{\mathrm{C}}}}}}}}_{{{{{{{\mathrm{i}}}}}}}}$$

The concentric radius (r_x_) as per Kiørboe [72] uses a diffusivity of N assumed to be 1.860 × 10^−5^ cm^2^ sec^−1^ and the background concentration of N (C_i_) is assumed to be negligible. Figure 5 presents the results for the two symbioses: *H. membranaceus* and *H. hauckii* at the two growth rates and as chains or singlets. Mean sinking rates for cells with a high form resistance (e.g., chains) are <10 m d^−1^ (Fig. 5). Simplified exudation calculations assuming no motility suggest that the phycosphere surrounding individual cells in these chains would range from ~3–70 nmol N L^−1^ at the cell surface and ~1–25 nmol N L^−1^ at a distance of twice the equivalent spherical radius (Fig. 6). Sinking would deform these plumes and increase flux away from the cellular boundary layer so these are considered maximal near-cell concentrations of exudate. More complex mechanistic models of diffusive fluid dynamics (e.g., x, y, z) would be needed to simulate deformation of exudate plumes as a function of sinking speeds and the flow field (laminar or turbulent) [73–75].

In oligotrophic regimes such as WTNA, where N concentrations in the surface mixed layer are often below detection, the symbiotically derived reduced N serves as an important source of new N to the surface ocean. Based on our calculations, we would predict the phycosphere surrounding these symbioses to be N-rich, and given sinking speeds <10 m d^−1^, these exudates retain the availability of limiting nutrients in the surface waters and fuel regional primary production rather than contributing to the mesopelagic nutrient inventory.

### Conclusions

Despite widespread distributions and occasional large-scale blooms, diatom-*Richelia* symbioses have been largely understudied. Our understanding of their metabolic activities and partner interactions has been limited to N_2_ fixation and the exchange of fixed N, respectively. Here, we report both the N_2_ and C fixation activity of single cells from three different diatom-*Richelia* symbiotic populations collected and assayed in the wild. A robust biometric relationship was identified where larger symbiotic cells have higher activity. N_2_ fixation by one symbiotic population appears to be light dependent, whereas unexpectedly C fixation is highly variable and independent of light. Inhibitor experiments designed to shut down the host photosynthesis and communication resulted in depressed C and N_2_ fixation activity suggesting that the hosts are the primary C fixing partner and likely control their symbionts N_2_ fixing activity. Single cell rates and estimated sinking velocities were combined with a simplified model to predict that most of the fixed N is released in the upper water column by the symbiotic diatoms. In summary, the observations reported here for both abundance and in situ activity contributes to an improved understanding of symbiotic diatom distribution, ecology, and contribution to N/C cycling.

## Supplementary information

Supplementary Materials

Supplementary Figure 1

Supplementary. Figure 2

Supplementary Table 1

Supplementary Table 2

Supplementary Table 3

Supplementary Table 4

Supplementary Figures Captions
