# Supplementary material for: The rate and fate of N2 and C fixation by marine diatom-diazotroph symbioses
Source: ISME J. 2021 Aug 24;16(2):477–87. doi: 10.1038/s41396-021-01086-7 (PMC8776783; doi:10.1038/s41396-021-01086-7)
Supplement: Supplementary file 1 — Supplementary Materials [file 41396_2021_1086_MOESM1_ESM.docx]

**Supplementary Materials**

*Microscopy observations for station selection.* At each station, an initial pre-dawn plankton net (Sea Gear student model 9100, 25 cm diameter, length 3:1; 80 μm mesh) was deployed by hand in the upper 1-5 m of the surface from the stern for 5-10 min. Subsequently the cod-end was poured into a larger container and diluted with approximately 5 L of surface seawater. A subsample (250-500 mL) of the concentrated plankton was gravity filtered onto a 10 μm pore size 47 mm membrane filter (Poretics; Millipore, Billerica, MA USA) using a standard filter tower (Millipore). The 47 mm filter was placed onto an oversized glass slide (75 mm x 50 mm x 1 mm) with one droplet of filtered seawater (FSW), coverslip applied and quickly scanned at 400X under a Zeiss Axioskop epi-fluorescence microscope (Zeiss, Berlin, Germany) fitted with blue (450-490 nm) and green (510-560 nm) excitation filter sets. Under blue excitation, the cyanobacterial symbiont filaments emit yellow/orange and are clearly distinguishable from the autofluorescence of the diatom chloroplasts (Suppl. Figure 1). Under green excitation, the cyanobacterial filaments emit a red-orange autofluorescence.

*Calculations: biovolume and biomass conversion, ^13^C and ^15^N assimilation, symbiotic N and C contribution to whole ^13^C and ^15^N assimilation.* The cell biovolume (μm3) of individual symbiotic cells was estimated as described previously using measurements of cell diameter, apical, and transapical axes (1). The initial carbon (C) content of the symbiotic diatoms was estimated using the Strathmann (2) equations, where biovolume (V) was used in place of plasma volume. The C content was then used to estimate nitrogen (N) content by assuming a Redfield ratio (C: N) of 6.6 (3). Since none of the symbiotic diatoms have been brought into pure culture for a standard elemental analysis, we considered our estimate of the initial C and N content based on biovolume and Redfield ratio as conservative and a reasonable alternative. The cell specific C and N2 assimilation (FC or FN) was calculated for each time point as follows:

FC = (13Cex × Ccon)/CSR (1)

FN= (15Nex × Ncon)/NSR (2)

where 13Cex and 15Nex is the mean of the 13C/12C and 15N/14N of the individual ROIs, respectively for a particular time point corrected for by the mean value of the respective ratios for whole water bulk analyses time 0 samples (see above) and divided by 100. The Ccon and Ncon are the initial C and N content, respectively, estimated as described above and CSR and NSR are the labeling percentage of 13C and 15N, respectively, in the experimental bottle. The assimilated C and N was divided by the incubation time (h) to determine the cell-specific C and N2 fixation rates (fmol C cell-1 h-1 or fmol N cell-1 h-1). The C-based and N-based growth rates were determined as follows:

V=1/t × ( R_F_-R_I_/R_S_-R_I_ ) (3)

where t is time in days, R_F_ is the estimated mean value of the ROIs at a specific time point in a particular cell type (symbiosis, host, vegetative cell), the R_I_ is the AT% (^13^C or ^15^N) in the time zero samples for the respective cell type, and R_S_ is the calculated AT% of ^13^C or ^15^N in the experimental bottle according to Montoya et al. (4).

To assess the contribution of the symbiotic diatom cells to the whole water (bulk) assimilation estimated by the EA-IRMS analyses, the bulk assimilation was divided by the mean N2 and C assimilation from nanoSIMS analyses normalized to the cell abundances for the symbiotic diatoms (determined by the microscopy cell counts from each time point).

All statistical analyses (t-tests, Mann-Whitney) were run in Excel and R software (5). Curve-fitting procedures for determining the relationship between irradiance (I) and N_2_ fixation rate (NFR) used a hyperbolic tangent model (6) fit in Matlab via nonlinear least squares:

NFR = NFR_max_ × tanh ((α_NFR_ × I)/ NFR_max_ (4)

where the maximum rate of N_2_ fixation (NFR_max_) was 3.1 fmol N cell^-1^ h^-1^ and initial slope of the rate-response curve (α_NFR_) was 0.044 fmol N cell^-1^ h^-1^ (μE m^-2^ s^-1^)^-1^). The half-saturation irradiance (E_K_= NFR_max_/ α_NFR_) is thus 70 μE m^-2^ s^-1^. The 95% confidence intervals for the model fit (shown in Figure 2) correspond to lower and upper bounds of NFR_max_ of 2.3-3.9 fmol N cell^-1^ h^-1^ and α_NFR_ of 0.018 – 0.07 fmol N cell^-1^ h^-1^ (μE m^-2^ s^-1^)^-1^. The adjusted r^2^ value for this fit is 0.56. Carbon fixation did not behave predictably as a function of light.

**References**

(1) Foster RA, Kuypers MMM, Vagner T, Paerl RW, Musat N, Zehr JP. Nitrogen fixation and transfer in open ocean diatom-cyanobacteria symbioses. *ISME J* 2011; **5**: 1484-1493.

(2) Strathmann RR. Estimating the organix carbon content of phytoplankton from cell volume or plasma volume. *Limnol Oceangr* 1967; **12**: 411-418.

(3) Redfield AC. On the proportions of organic derivations in seawater and their relation to the composition of plankton. In: Daniel RJ (ed). *James Johnstone Memorial Volume*. University Press Liverpool: UK, pp 177-192.

(4) Montoya JM, Voss M, Kähler P, Capone DG. A simple, high-precision, high sensitivity tracer assay for N_2_ fixation. *Appl Environ Microbiol* 1996; **62(3)**: 986-993.

(5) R Core Team. R: A language and environment for statistical computing. R Foundation for Statistical computing. Vienna, Austria, http://www.R-project.org.

(6) Jassby AD, Platt T. Mathematical formulation of the relationship between photosynthesis and light for phytoplankton. *Limnol Oceangr* 1976; **21(4)**: 540-547.
