# Supplementary figures and images for: The rate and fate of N2 and C fixation by marine diatom-diazotroph symbioses
Source: ISME J. 2021 Aug 24;16(2):477–87. doi: 10.1038/s41396-021-01086-7 (PMC8776783; doi:10.1038/s41396-021-01086-7)

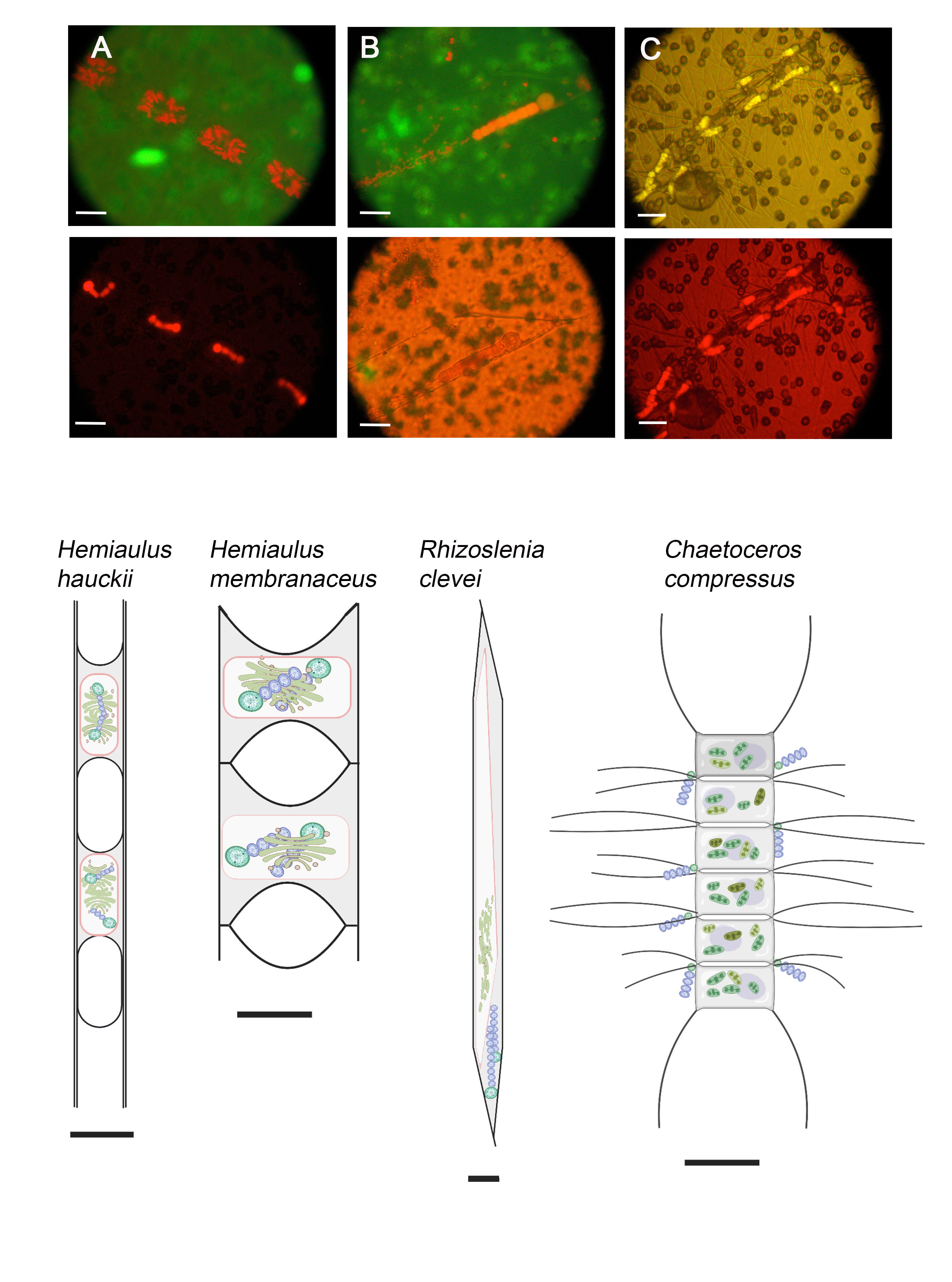

Supplement: Supplementary file 2 — Supplementary Figure 1 [file 41396_2021_1086_MOESM2_ESM.jpg]

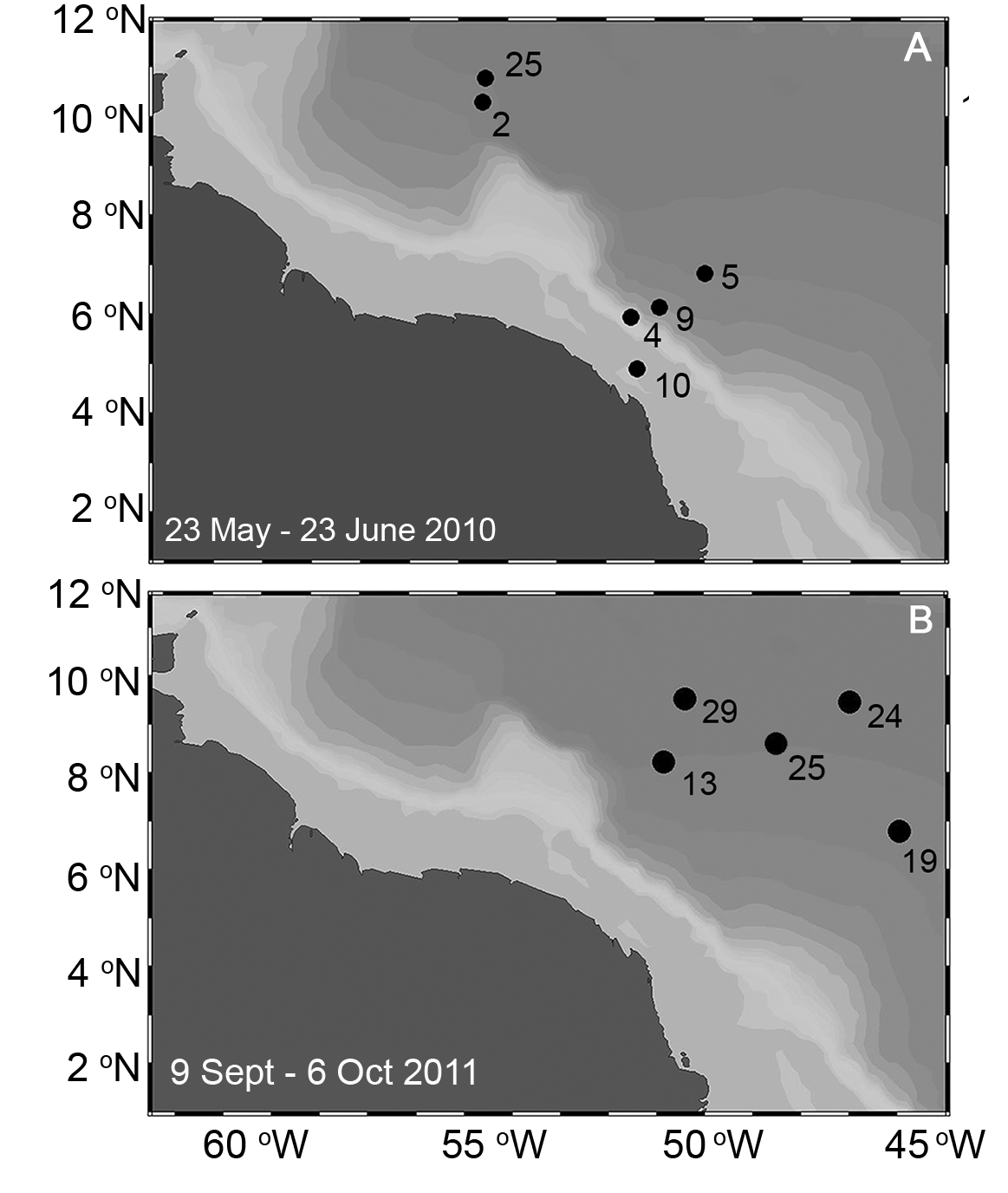

Supplement: Supplementary file 3 — Supplementary. Figure 2 [file 41396_2021_1086_MOESM3_ESM.tif]
