# Supplementary material for: The rate and fate of N2 and C fixation by marine diatom-diazotroph symbioses
Source: ISME J. 2021 Aug 24;16(2):477–87. doi: 10.1038/s41396-021-01086-7 (PMC8776783; doi:10.1038/s41396-021-01086-7)
Supplement: Supplementary file 8 — Supplementary Figures Captions [file 41396_2021_1086_MOESM8_ESM.docx]

Supplementary Figure 1. Epi-fluorescent and light micrographs (blue, 459-490 nm; green: 510-560 nm) of the wild diatom-cyanobacterial symbioses imaged in the field. A schematic (bottom) showing the continuum of symbiont cellular location in the various diatom-cyanobacteria symbioses: internal, partial and external. A. A chain of *Hemiaulus hauckii*-*Richelia* symbioses imaged under blue excitation and white light. Below is the corresponding image under green excitation. Note in the top image, *Richelia* are not easily visible, while with green excitation, filaments of *Richelia* emit red and are conspicuous. B. Blue excitation of the apical end of symbiotic *Rhizosolenia* spp. with one filament of *Richelia.* Below is the corresponding green excitation image taken with white light to show the diatom outer silica frustule boundary. The inner cell membrane of the diatom host is not visible. C. Blue (top) and green (bottom) excitation micrographs of a *Chaetoceros compressus* diatom with numerous *Calothrix* epibionts attached to its outer surface. Note that the point of attachment is often with the heterocyst, and the diatom appears devoid of chloroplasts. Scale bars are approximately 10 μm.

In the schematic drawings, the outer silica membrane (frustule) of the diatom host is outlined in black, while inner diatom cell membrane (plasmalemma) in *Hemiaulus* spp. and *Rhizosolenia cleveii* is shown in pink; diatom chloroplasts are green. *Richelia* filaments (trichomes) are illustrated as chains of vegetative cells (blue) with terminal heterocysts (green). *Richelia* are true endobionts in the *Hemiaulus* spp. penetrating the diatom cytoplasm, while they remain partially integrated in the *Rhizosolenia* spp. diatoms and reside between the host frustule and inner cell membrane. The *Calothrix* symbionts of *Chaetoceros* attach to the outside of the diatoms. Typically, two shorter *Richelia* filaments (3-4 vegetative cells) are observed in *Hemiaulus* spp., while the number and length of *Richelia* filaments can vary in *Rhizosolenia* spp. *Calothrix* filaments are also short (3-4 vegetative cells), and the numbers of symbionts attach can also vary. Note the difference in host dimensions for the two *Hemiaulus* spp. Scale bar is approximately 20 μm. Schematic has been made in Biorender.

Supplementary Figure 2. Map of the WTNA with stations where incubations were performed in 2010 (A) and 2011 (B). The map was generated in Ocean Data View software.
